# Characterization of an Isotype-Dependent Monoclonal Antibody against Linear Neutralizing Epitope Effective for Prophylaxis of Enterovirus 71 Infection

**DOI:** 10.1371/journal.pone.0029751

**Published:** 2012-01-18

**Authors:** Xiao Fang Lim, Qiang Jia, Wei Xin Khong, Benedict Yan, Balraj Premanand, Sylvie Alonso, Vincent T. K. Chow, Jimmy Kwang

**Affiliations:** 1 Animal Health Biotechnology, Temasek Life Sciences Laboratory, Singapore; 2 Department of Microbiology, Yong Loo Lin School of Medicine, National University of Singapore, Singapore; 3 Immunology Programme, Yong Loo Lin School of Medicine, National University of Singapore, Singapore; 4 Graduate School for Integrative Sciences and Engineering, National University of Singapore, Singapore; 5 Department of Pathology, National University Health System, Singapore; University of Sao Paulo, Brazil

## Abstract

**Background:**

Enterovirus 71 (EV71) is the main causative agent of Hand, Foot and Mouth disease (HFMD) and is associated with severe neurologic complications and mortalities. At present, there is no vaccine or therapeutic available for treatment.

**Methodology/Principal Finding:**

In this study, we generated two mAbs, denoted as mAb 51 and 53, both targeting the same linear epitope on VP1 capsid protein, spanning amino acids 215–219. In comparison, mAb 51 belonging to isotype IgM possesses neutralizing activity *in vitro*, whereas, mAb 53 belonging to isotype IgG1 does not have any neutralizing ability, even towards its homologous strain. When mAb 51 at 10 µg/g of body weight was administered to the 2-week-old AG129 mice one day prior to lethal challenge, 100% *in vivo* passive protection was observed. In contrast, the isotype control group mice, injected with an irrelevant IgM antibody before the challenge, developed limb paralysis as early as day 6 post-infection. Histological examination demonstrated that mAb 51 was able to protect against pathologic changes such as neuropil vacuolation and neuronal loss in the spinal cord, which were typical in unprotected EV-71 infected mice. BLAST analyses of that epitope revealed that it was highly conserved among all EV71 strains, but not coxsachievirus 16 (CA16).

**Conclusion:**

We have defined a linear epitope within the VP1 protein and demonstrated its neutralizing ability to be isotype dependent. The neutralizing property and highly conserved sequence potentiated the application of mAb 51 and 53 for protection against EV71 infection and diagnosis respectively.

## Introduction

Enterovirus 71 or EV71 (BrCr strain) was first isolated and identified in the United States in 1969 [1] , and was not associated with hand, foot mouth disease (HFMD) until 1973, when small epidemics broke out in Japan and Sweden [2], [3]. From then on, successive waves of EV71 outbreaks have been reported globally, in United Kingdom, Australia, Sweden, Bulgaria, Japan, China, Hong Kong, Taiwan, Malaysia and Singapore [2], [4], [5], [6], [7], [8], [9], [10]. Over the past decade, the Asia-Pacific region was considered the most seriously affected area, with occurrence of both major and small-scale outbreaks associated with mortalities and neurologic complications such as aseptic meningitis, fatal encephalitis and poliomyelitis-like paralysis [11]. In the 1998 outbreak in Taiwan, EV71 infected thousands of children and resulted in 405 severe cases of neurologic disease, and 78 deaths in children [10], [12]. HFMD had also emerged in China since 2008, resulting in approximately 3.4 million of accumulated cases with 1400 fatalities [13]. Thus, EV71 represented a pre-eminent neurotropic virus ever since the almost complete eradication of poliomyelitis.

Typical of a member of the family *Picornaviridae*, EV71 is a small, non-enveloped, positive-stranded RNA virus, comprising four capsid proteins VP1, VP2, VP3 and VP4. These capsid proteins form the icosahedral structure, with VP1–3 exposed on the virus surface and VP4 arranged internally [14]. Among these capsid proteins, VP1 is believed to be the major contributor in viral pathogenesis, playing critical roles in the adsorption and uncoating processes of virus infection [15]. In addition, VP1 protein also contains important neutralization sites. Studies utilizing high-titer human neutralizing antibodies from cord blood samples collected in Sarawak in 1999 demonstrated that these neutralizing antibodies were more reactive towards the N-terminal half of VP1 protein [16]. In contrast, another study revealed linear neutralizing epitopes on VP1 protein [17], making VP1 a good candidate for subunit vaccine. EV71 can be divided into 3 genotypes namely A, B and C. Genotypes B and C can be further divided into subgenogroups denoted as B1–5 and C1–5 respectively. These genotypes and subgenogroups are designated based on their VP1 gene sequences [18].

Up till now, health authorities can only rely on public health surveillance and quarantine to prevent and control the spread of the virus in the case of an outbreak, as EV71-specific vaccines or therapeutics are not yet routinely available. Intravenous immunoglobulin (IVIG) is produced by extracting and pooling together human immunoglobulin [19]. It has been employed as a therapeutic agent that can effectively abolish or modify a variety of infectious and inflammatory diseases such as Japanese encephalitis, West Nile virus encephalitis and coxsackievirus infection [19], [20], [21]. This method has also been used prophylactically and therapeutically against enterovirus infection in neonates and immunocompromised adults [22], [23]. For instance, IVIG is used extensively in the management of EV71 outbreaks in Taiwan, Western Australia and also in China [24]. However, preparation of a concentrated, biologically active, and safe immune globulin for human use involves a complex immunochemical method [19]. There are also issues pertaining to risks in transmission of other human pathogens. Therefore, monoclonal antibodies (mAb) offer a better alternative treatment, and have indeed attracted much attention from the pharmaceutical industry recently. In view of this, we are interested in developing anti-EV71 monoclonal antibodies which possess neutralizing ability against all EV71 genotypes, to facilitate the study of their potential applications for preventation and infection treatment.

In this study, we generated two mAb (designated mAb 51 and 53) by immunization of inactivated whole virus of EV71 strain NUH0083-B5. The mAbs were fully mapped, and found to target the same linear epitope on VP1 capsid protein, spanning amino acids 215–219. However, they belonged to different immunoglobulin isotypes and also displayed different neutralizing ability against EV71 infection. We demonstrated that mAb 51 (belonging to isotype IgM) possessed neutralizing activity *in vitro* against all EV71 genotypes, and also conferred 100% passive protection *in vivo* against EV71 infection prophylactically. In contrast, mAb 53 (belonging to isotype IgG1) did not possess any neutralizing ability both *in vitro* and *in vivo*.

## Results

### Identification of specific mouse mAb against EV71

**Figure 1:**
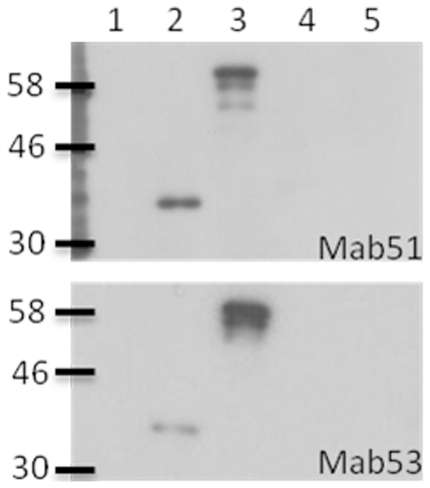
Characterization of Mab 51 and 53 by western blot hybridization. Lane 1: RD Rhabdomyosarcoma cells total cell lysate. Lane 2: Concentrated C4 virus. Lane3: GST-tagged VP1 recombinant protein (∼61 kDa). Lane4: GST-tagged VP2 recombinant protein (∼56 kDa). Lane 5: GST-tagged VP3 recombinant protein (∼50 kDa). mAb 51 and 53 were applied on respective membrane to test for their immunoreactivity.

EV71 strain NUH0083-B5 was propagated and concentrated from virus-infected rhabdomyosarcoma (RD) cells, and subjected to BEI inactivation before injection into BALB/c mice. The anti-sera raised against EV71 were analyzed by IFA, to test for immunoreactivity of the anti-sera with EV71 virus. Mice were subsequently sacrificed for cell fusion to generate hybridomas secreting EV71-specific mAbs. The positive clones secreting EV71-specific mAbs were identified by IFA, and selected for subcloning by limiting dilution. Imumunospecificities of mAb 51 and 53 were assayed against total viral proteins and recombinant capsid proteins (VP1, VP2 and VP3) by Western blotting. In Figure 1, both mAb 51 and 53 reacted in the same manner, with a single band observed in lane 2 (concentrated C4 virus) at an apparent molecular mass of ∼32 kDa. Its size corresponded to the VP1 capsid protein (32.7 kDa). A single band with an apparent size of 58 kDa could also be observed in lane 3 which was loaded with total bacterial cell lysate expressing GST-tagged VP1 recombinant protein. Its size corresponded to the size of GST-tagged VP1 protein (58 kDa). These data indicated that mAb 51 and 53 were specific for VP1 protein, and did not cross-react with other capsid or viral proteins. Being able to react with the VP1 protein under denaturing conditions implied that the mAbs targeted linear epitopes.

### Epitope mapping of mAb 51 and 53

**Figure 2:**
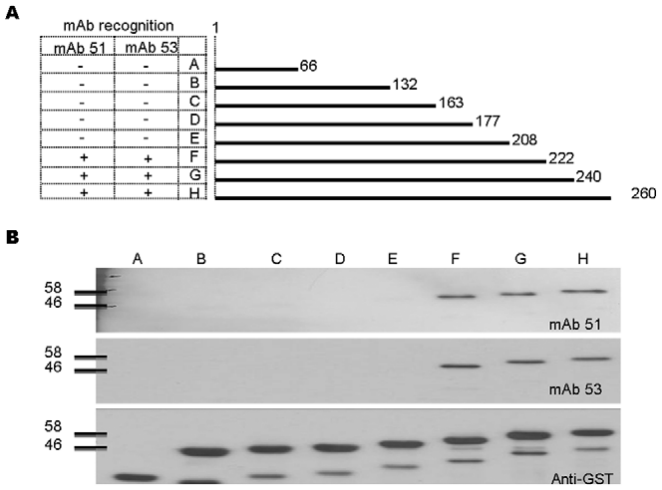
Initial epitope mapping of mAb 51 and 53 by Western blot analysis. (A) C-terminal truncated fragments of VP1 protein (A–H), with peptide-spanning regions depicted. (B) Expression of protein fragments determined with mAb against GST protein. Western blot results indicated interaction of both mAb 51 and 53 with fragment F–H, implying the location of the epitope within amino acids 208–222.

C-terminal truncated VP1 proteins were expressed as GST-fusion proteins by cloning the corresponding fragments into pGEX-4T-1 as described earlier. Respective recombinant plasmids were transformed into *E. coli* cells, and induced for protein expression. As depicted in Figure 2A, a total of eight C-terminal truncated protein fragments were expressed. Fragments A (1–66), B (1–132), C (1–163), D (1–177), E (1–208), F (1–222), G (1–240) and H (1–260) were successfully expressed and detected with anti-GST mAb as shown in Figure 2B. Western blots also showed that mAb 51 and 53 were only reactive to protein fragments F, G and H implying that epitopes of both mAb were located within amino acids 208–222 of VP1 protein.

**Figure 3:**
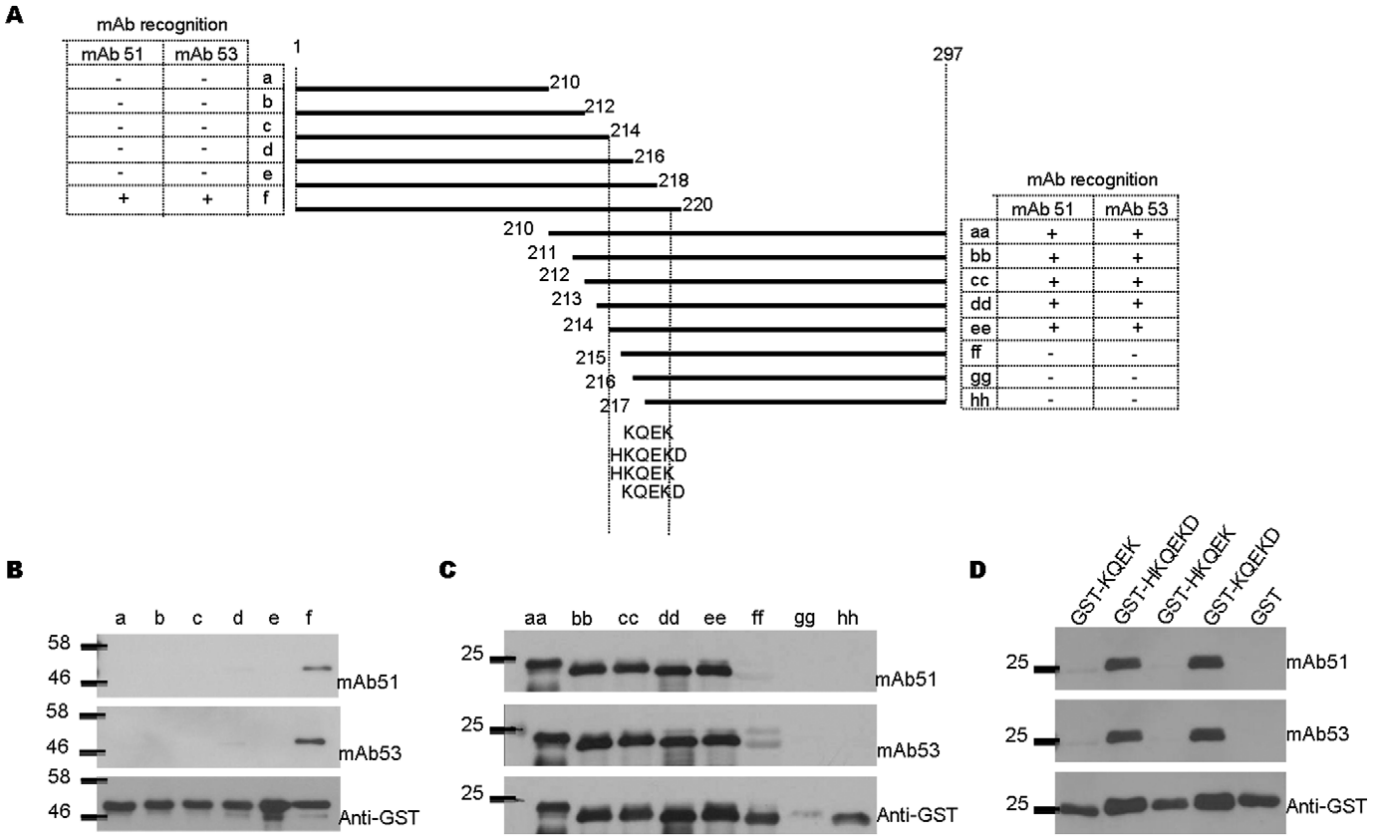
Detailed epitope mapping of mAb 51 and 53 by Western blot analysis. (A) C-terminal truncated fragments of VP1 protein (a–f), N-terminal truncated fragments of VP1 protein (aa–hh), and GST-(epitope) peptide spanning regions as depicted. Expression of protein fragments was determined with mAb against GST protein. (B) Western blot results of C-terminal truncated proteins (a–f) indicated interaction of both mAb 51 and 53 with fragment f only, implying that the last amino acid of the epitope should be at amino acid 219 or 220. (C) Western blot results of N-terminal truncated proteins (aa–hh) indicated that the first amino acid of the epitope should be amino acid 214. Thus, the putative epitope of mAb 51 and 53 should span amino acid 214 to either 219 or 220. (D) Western blot analysis of GST-putative epitope proteins (GST-KQEK, GST-HKQEKD, GST-HKQEK, GST-KQEKD) and GST as negative control. Interaction with both GST-HKQEKD and GST-KQEKD indicated that the epitope of mAb 51 and 53 is KQEKD (215–219).

The C-terminal truncated proteins were then expressed from amino acids 1 to 220, and with additional two amino acids for each of the consecutive fragments at the C-terminal. Figure 3A shows the schematic presentation of the protein fragmentation. In Figure 3B, mAb 51 and 53 specifically recognized only fragment f(1–220), indicating that the last amino acid of the epitope was either at amino acid position 219 or 220. Another set of N-terminal truncated proteins expressed from amino acids 210 to 297, and with deletion of one amino acid for each of the subsequent fragments at the N-terminal as depicted in Figure 3A were expressed to identify the first amino acid of the epitope. Figure 3C shows that mAb 51 and 53 recognized fragment aa-ee, indicating that the first amino acid of the epitope should be at amino acid position 214. With the Western blot assays, we deduced that the epitope should span amino acids 214–219 or 214–220. Subsequently, we expressed four putative epitopes fused with GST protein, i.e. GST-KQEK, GST-HKQEKD, GST-HKQEK, and GST-KQEKD. Figure 3D shows that mAb 51 and 53 specifically recognized only GST-HKQEKD and GST-KQEKD only, indicating that the epitope of mAb 51 and 53 should KQEKD, spanning amino acids 215 to 219 within the VP1 capsid protein.

**Figure 4:**
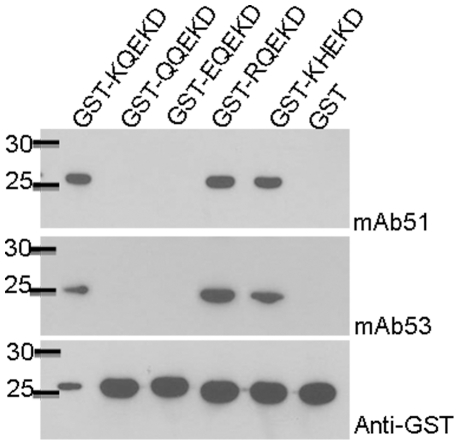
Evaluation of interactions of mAb 51 and 53 with mutated epitopes. Mutated epitopes (QQEKD, EQEKD, RQEKD and KHEKD) were expressed as GST-fusion proteins. Expression of proteins was determined with mAb against GST. mAb interacted with the authentic epitope (KQEKD) and mutated epitopes (RQEKD and KHEKD). They failed to recognize the mutated epitopes QQEKD and EQEKD.

The epitope KQEKD was subjected to protein-protein BLAST analysis against all enterovirus sequences in the GenBank, and three single amino acid mutations at the first amino acid of the epitope were identified in EV71. Lysine (K) was mutated into glutamine (Q), glutamic acid (E) and arginine (R). Two of such mutated sequences were found for each of the mutation. Mutations of K to Q and E occurred in the EV71 sequences from China reported from 2008 to 2009, while mutations of K to R were found in EV71 sequences from Korea (2008) and Japan (2004). Another mutation at the second amino acid from glutamine (Q) to histidine (H) were also identified. In order to evaluate the interactions of our mAbs with these epitopes, we expressed the mutated epitopes with the GST tag. Figure 4 clearly illustrates that mAb 51 and 53 were not capable of recognizing the mutated epitopes QQEKD and EQEKD. However, they could recognize the other two mutated epitopes, i.e. RQEKD and KHEKD.

### Specificity of mAb 51 and 53 to EV71 subgenogroups but not to coxsackievirus A16

The epitope of mAb 51 and 53 was found to be highly conserved and specific for EV71 strains. According to the BLAST results, the mAb can recognize all EV71 strains except the four sequences with mutations from K to Q and E. In addition, the exact epitope was absent in all other enteroviruses such as coxsackievirus A16, another important causative agent of HFMD. The cross- reactivity of mAb 51 and 53 was tested against 11 heterologous EV71 strains as well as coxsackievirus A16 by Western blot assay and IFA.

**Figure 5:**
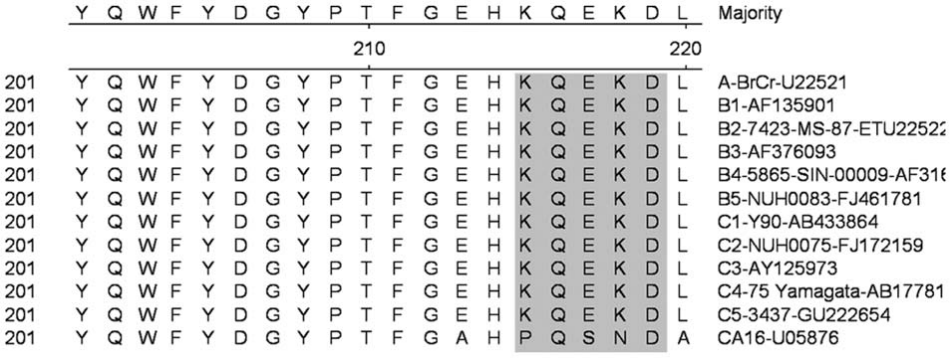
Partial alignment of heterologous EV71 strains from all representative subgenogroups and coxsackievirus 16 based on the VP1 amino acid sequences. Alignment of the epitope KQEKD was highlighted in grey, with 100% homology with all representative EV71 subgenotypes. A different epitope was observed in CA16 at amino acid position 215–219 of VP1 protein.

**Figure 6:**
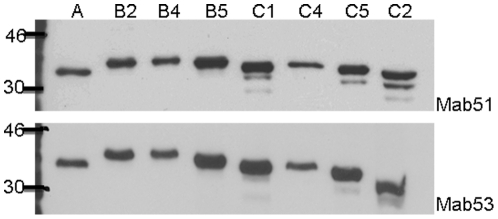
Cross reactivity of mAb 51 and 53 with concentrated EV71 viruses. Concentrated EV71 strains of various subgenogroups (A, B2, B4, B5, C1, C4, C5 and C2) were separated on SDS-PAGE. A single band of approximately 32 kDa was detected in all the viruses of different subgenogroups. mAb 51 and 53 were able to cross-react with all the EV71 strains tested. A slight variation of VP1 protein size was also observed among the different EV71 subgenotypes.

**Figure 7:**
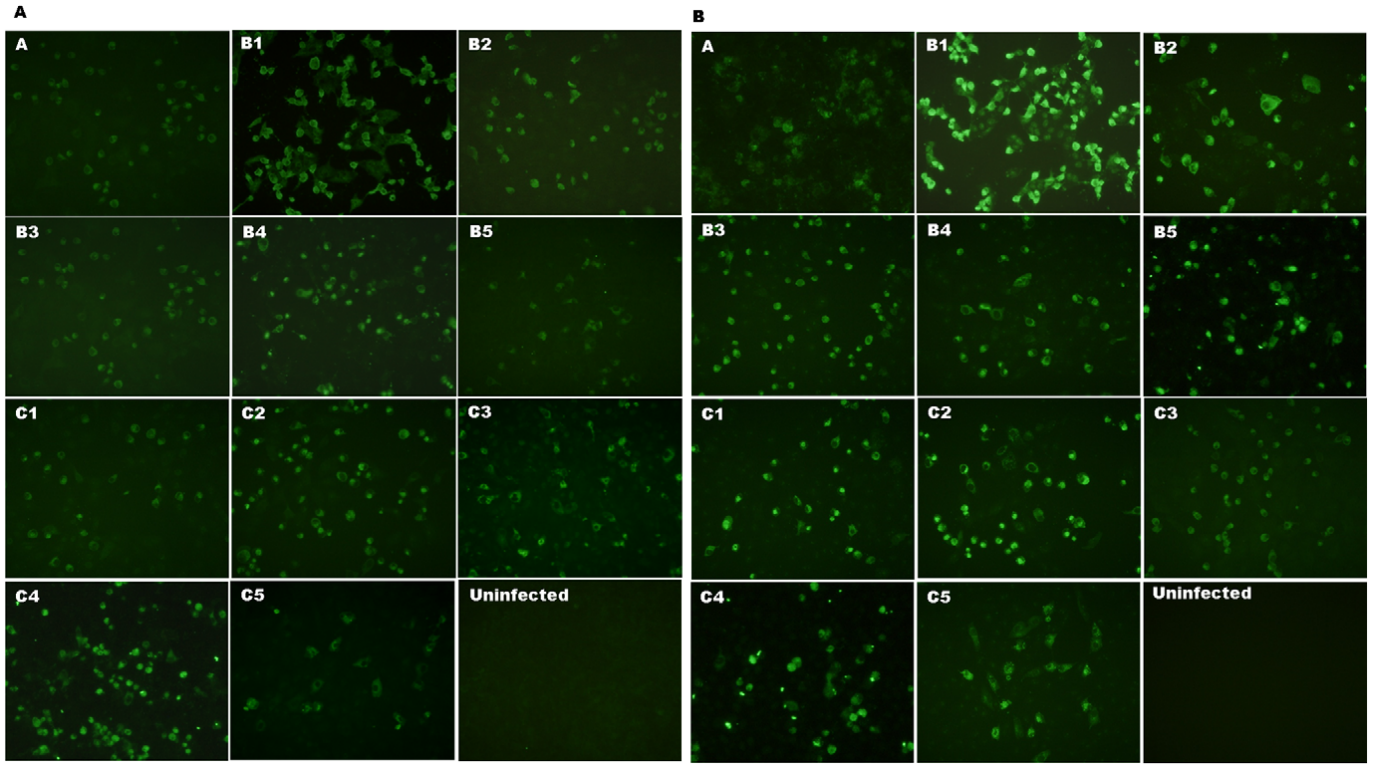
IFA of Vero cells infected with heterologous strains of EV71 with mAb 51 and 53. (A) mAb 51 and (B) mAb 53 cross-reacted with all the 11 representative EV71 strains (depicted on the top-left corner of each image). Immunofluoresent signals observed in the infected Vero cells as well as in apoptotic cells indicated cross-reactivity between EV71 subgenotypes. Uninfected Vero cells served as negative control.

Figure 5 shows the partial alignment of VP1 amino acid sequences of all EV71 subgenogroups (A, B1–B5, C1–C5 and CA16). Epitope KQEKD (highlighted in grey) was found to be fully conserved among the 11 representative strains of different EV71 subgenogroups. In contrast, the epitope was found to be rather different in CA16. Figure 6 confirmed that mAb 51 and 53 were able to cross-react with 8 representative EV71 subgenogroups (A, B2, B4, B5, C1, C2, C4 and C5). A single band of approximately 32 kDa which corresponds to VP1 protein was observed. A slight variation of the VP1 protein size was also observed among the different EV71 strains. In addition, Figure 7 further illustrates that mAb 51 and 53 were able to cross-react with all the 11 representative EV71 strains which were used for the alignment analysis by IFA. With these supporting data, we could confirm that mAb 51 and 53 are capable of cross-reacting with all EV71 subgenogroups.

**Figure 8:**
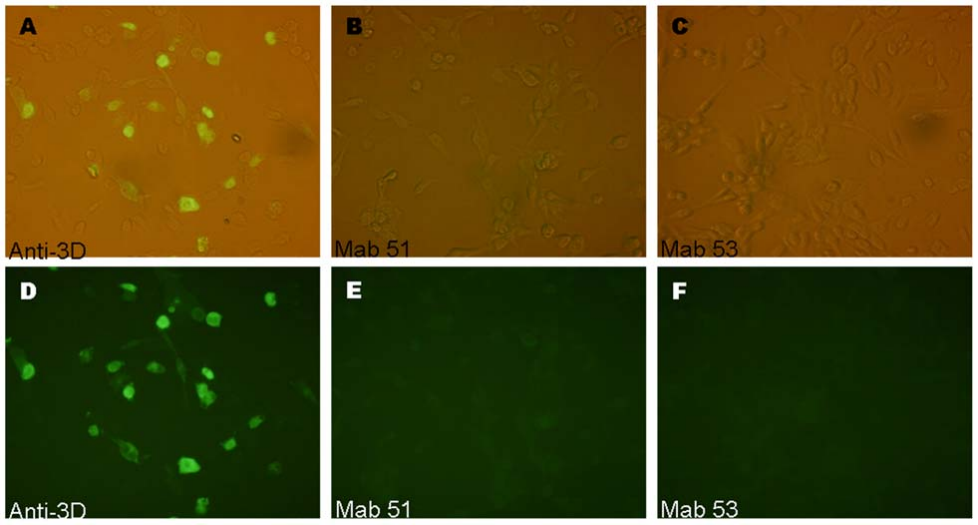
IFA of RD cells infected with CA16 virus. (A–C) IFA images were presented in bright field. (D–F) IFA images presented in fluorescence. (A&D) Immunofluoresence signals were observed with mAb against 3D polymerase of EV71. (B&E) No immunofluoresence signal observed with mAb 51. (C&F) No immunofluoresence signal observed with mAb 53. Absence of immunofluoresence signal with mAb 51 and 53 indicated no cross-reactivity with CA16.

In determining whether mAb 51 and 53 can distinguish between EV71 and coxsackievirus A16 (CA16) infections, RD cells were infected with CA16 and IFA was conducted with mAb 51 and 53 (Figure 8). In that IFA, another mAb 4B12 directed against the 3D polymerase protein was included as a positive control to determine the infection of RD cells by CA16. The mAb was found to be able to cross- react with CA16 owing to their common epitope (unpublished data). We only observed signal in Figure 8A and 8D where infected cells were recognized by mAb 4B12. However, no signals were observed with mAb 51 and 53 as demonstrated in Figure 8B, 8C, 8E and 8F. Hence, we concluded that our mAb did not cross react with the representative strain of CA16. The result also corresponded with the protein alignment results in Figure 5, where a rather different epitope was found in CA16. This indicated that mAb 51 and 53 can distinguish between EV71 and CA16 infections.

### *In vitro* neutralizing activity of mAb 51 but not mAb 53

In testing whether mAb 51 and 53 have neutralizing activities against homologous and heterologous strains of EV71, supernatants of the hybridomas were tested by the *in vitro* microneutralization assay. RD cells were infected with 200 tissue culture infective dose (TCID_50_) of representative strains from all subgenogroups as listed in Table 1. Complete protection from cytopathic effects (CPE) could be observed in RD cells when mAb 51 was applied. Ascites derived from mAb 51 provided a neutralizing titer of about 1024, while the hybridoma supernatant showed a titer of 32 against homologous and heterologous strains of EV71. However, mAb 53 did not display any neutralizing activity even against its homologous B5 strain. Despite sharing a common epitope, only mAb 51 possessed neutralizing ability. In characterizing the immunoglobulin isotype of mAbs, mAb 51 was found to be isotype IgM, and mAb 53 to be isotype IgG1. The results suggested that the immunoglobulin isotype may play a crucial role in the neutralization ability of the mAb, in the context of epitope KQEKD.

**Table 1:** Representative strains of EV71 subgenotypes.

Name	Accesion number	Subgenotypes
**BrCr**	U22521	A
**RG EV71-VP1(B1)**	AF135901	B1
**7423/MS/87**	ETU22522	B2
**RG EV71-VP1(B3)**	AF376093	B3
**HFM41**	AF316321	B4
**NUH0083**	FJ461781	B5
**Y90-3761**	AB433864	C1
**NUH0075**	FJ172159	C2
**RG EV71-VP1(C3)**	AY125973	C3
**75-Yamagata**	AB177813	C4
**3437/SIN/06**	GU222654	C5

### Passive protection against lethal EV71 strain in AG 129 mice

A novel mouse model of EV71 infection was used to test the *in vivo* protective efficacy of the antibodies. In the mouse model, 2-week-old immunodeficient AG129 mice were found to be susceptible to the infection with the non mouse-adapted EV71 strain HFM 41 via intraperitoneally (i.p.) route of inoculation. The infected AG129 mice displayed progressive neurological manifestations such as limb paralysis prior to death (unpublished data).

In the experiment, 2-week-old AG129 mice were injected i.p. with mAb 51 at 10 µg/g of body weight one day prior to lethal challenge with 10^7^ PFU of EV71 strain HFM 41. An isotype control group was injected with an irrelevant mouse IgM antibody (isotype control) before challenge. The control animals which received an isotype antibody developed severe limb paralysis as early as day 6 post-infection, while the mice pre-treated with mAb51 did not display any of the disease manifestations and remained healthy throughout the experiment. Our result thus suggested that the anti-EV71 antibody mAb 51 was able to achieve 100% protection against the lethal EV71 challenge at a dose of 10 µg/g of body weight.

### Histopathologic examination of spinal cords of mice

**Figure 9:**
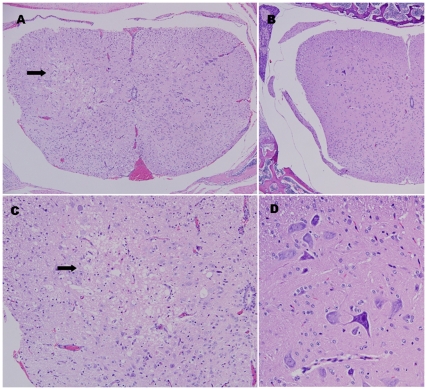
Histopathology of spinal cord of EV71-infected AG129 mice and prophylactically mAb-protected mice. (A) Cross-section of spinal cord from isotype control mice with arrow pointing to region with neuropil vacuolation and neuronal loss without inflammation in the anterior horn (at original magnification of 100×). (C) Higher power view of Figure 9A at original magnification of 200×. (B) Cross-section of spinal cord from prophylactically mAb-protected mice showing no significant pathology (at original magnification of 100×). (D) Higher power view of Figure 9B showing no significant pathology (at original magnification of 400×).

**Figure 10:**
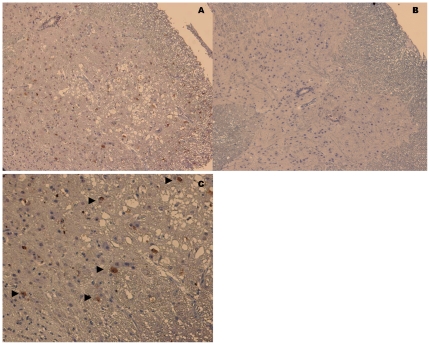
Immunohistochemistry of spinal cord of isotype control mice and prophylactically mAb-protected mice. (A) Cross section of spinal cord from isotype control mice with positive staining from mAb against 3D polymerase in the virus infected cells. (C) Higher power view of Figure 10A at original magnification of 400×. Arrow pointing to virus infected cells (B) Cross section of spinal cord from ly mAb-protected mice with no significant pathology (at original magnification of 200×).

Histopathologic examination of mice from isotype control group revealed neuropil vacuolation and neuronal loss without inflammation in the anterior horn in the spinal cord (Figure 9A and 9C). The presence of EV71 infiltration into the spinal cord could also be detected by mAb 4B12 (Figure 10A and 10C) and mAb 53 (results not presented). In contrast, we did not observe such pathologic changes in mice from the prophylactic group. The intact spinal cord morphology (Figure 9B, 9D and 10B) suggested that mAb 51 was capable of conferring *in vivo* passive protection against EV71 infection.

### EV71 detection by quantitative reverse transcription polymerase chain reaction (qRT-PCR)

**Figure 11:**
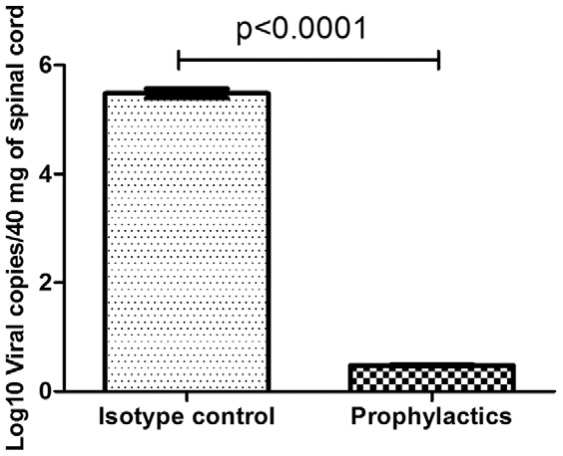
Analysis of EV71 viral copies by real-time PCR. Each column represents the mean of triplicate assay with standard deviation. Spinal cord from isotype control mice in the 7 dpi and prophylactically mAb-protected mice in 20 dpi.

The concentrations of the viral RNA copies were found to be greater than 1×10^5^ copies/ml in isotype control (IgM) treated mice, whereas in the prophylactics group, we observed lower than 10 copies of viral RNA/ml (Figure 11).

## Discussion

Frequent HFMD epidemics caused by EV71 infection have created great public awareness and concerns over the past decade, especially in the Asia-Pacific region. Despite tremendous research efforts, there is still a lack of effective vaccine and treatment strategies against EV71 infections. IVIG has been employed extensively in regions such as Taiwan and China for the management of EV71 infections during outbreaks [24]. However, this approach may harbor the risks of transmission of infectious agents, and may suffer from inconsistency in batch preparations. We were motivated to study and develop a mAb which can confer similar protection against EV71 infection. The major advantage of using mAb over IVIG is its potential availability in large quantities, renewable and of constant quality. In our study, we immunized mice with inactivated whole EV71, and derived with two mAbs, i.e. mAb 51 and 53.

These mAb were mapped by recombinant peptides and found to recognize a common epitope spanning amino acids 215 to 219 within the VP1 capsid protein. The identified epitope is located within the SP70 synthetic peptide (spanning amino acid residues 208–222) which was previously reported to harbor a linear neutralizing epitope within homologous and heterologous EV71 strains [17]. However, the exact epitope within the 14 amino acid peptide was not reported. Prior to our studies, there has been report of mAb (IgG) reactive towards to SP70 peptide. However, they only illustrated *in vitro* neutralization, without further evaluation on its exact epitope and *in vivo* neutralization [25].

Ascites derived from mAb 51 was capable of neutralizing all representative EV71 strains up to the highest dilution of 1∶1024 *in vitro*, while mAb 53 did not show any neutralizing ability even against its homologous strain (NUS0083-B5). Isotyping of these mAb revealed that mAb 51 belonged to isotype IgM, while mAb53 belonged to IgG1. This suggested that immunoglobulin isotypes may play a crucial role for this particular epitope in its protective property against EV71 infection.In a recent study from Taiwan, it was demonstrated that a mAb (isotype IgG2a) which was reactive to VP1 peptide (spanning amino acid residues 211–220), only carried a neutralization titer of 1∶64 and exclusively against its homologous strain at a high concentration of 1 mg/ml, relative to the concentration ascite [26]. Thus, the difference in isotype has once again demonstrated variation in the neutralization activity. Another study in China has also reported on a mAb with neutralizing activity up to a dilution of 1∶64. However, the mAb possessed a different epitope from mAb 51 and its isotype was not mentioned [27].

IgM is the first antibody isotype produced by the humoral immune response, and exists as a pentamer with ten antigen-binding sites. Thus, IgM can simultaneously binds to multivalent antigens, contributing to an overall high avidity which is an combined synergistic strength of single bond affinities [28]. The importance of IgM in the neutralization of influenza virus by steric hindrance has been suggested long before [29]. Other mechanism such as aggregation of virus may be responsible for reduction of viral infectivity *in vivo* [30]. In addition, IgM antibodies specific against West Nile virus and *Nocardia brasiliensis* were also shown to play important roles in providing protection against infection [31], [32], [33]. Experiments could be done to unveil the mechanistic role of mAb 51 in neutralizing EV71 infection which can enhance the understanding of the protective role of Ig M in a typical EV71 infection.

mAb 51 was able to prophylactically confer 100% *in vivo* passive protection in mice against heterologous B4 strain. Histopathologic examination of the mice challenged with EV71 displayed neuropil vacuolation and neuronal loss at the anterior horn of the spinal cord. EV71 virus could also be detected by mAb 53 and 4B12 by immunohistochemistry. These pathologic changes and detection of EV71 virus were not observed in the mAb-protected mice. This suggested that mAb 51 could possess protective effects against EV71 infection. The neutralizing ability of mAb 51 is comparable with the antiserum elicited by the SP70 peptide which exhibited complete protection from CPE *in vitro* and 80% protection against EV71 infection *in vivo* [17], [34]. In the previous study, the IgG response induced by SP70 is as high as that induced by inactivated virus, suggesting that the neutralizing activity is attributed to IgG. Nevertheless, there is also a possibility that the protective effect may be due to the IgM present in the polyclonal antiserum. In a recent study on the performance of detecting IgM antibodies against EV71 for early diagnosis, it was reported that the detection rate of IgM is 95–100% for infected individuals after a month from onset and can still be detected even 40 days after the onset of infection [35]. The antiserum raised against SP70 collected 7 weeks after the first immunization may contain significant EV71-specific IgM levels which may confer protective effects against EV71 infection. However, there may also be other neutralizing epitopes within the SP70 peptide that contribute to the neutralizing activity in the antiserum raised against SP70. Therefore, additional experiments could be conducted to produce polyclonal antibodies against SP70, and then separating the population of IgM and IgG isotypes to separately evaluate their neutralizing ability.

Humanized mAb 51 might offer an alternative reagent for prevention during the management of a sudden EV71 outbreak before identification of another better candidate. This particular epitope carries neutralizing ability in the form of IgM. However, IgG is conventionally the preferred class for therapeutics antibodies due to its longer half life *in vivo* [36]. Therefore, more neutralizing mAbs, preferably IgG can also be discovered by producing human mAbs using transgenic mice or phage display in the near future.

BLAST analyses of the KQEKD epitope revealed that it is fully conserved among all EV71 strains but not in CA16. This result was supported by the Western blot and IFA data. This makes mAb 53 a promising candidate for the development of a rapid diagnostic kit as it is only specific for EV71 infection but not CA16. Comparatively, mAb 53 of IgG isotype is more suitable for this application due to its stability and ease in purification.

In conclusion, we have successfully generated two mAbs, and have defined the exact neutralizing epitope within the SP70 peptide. We have discovered that this linear neutralizing epitope is only effective for EV71 protection in the form of IgM, and may confer similar protection as the immune sera derived from SP70-immunized mice. Humanization of mAb 51 has potential application for prevention of EV71 infections. Given that it possesses a highly conserved epitope among all EV71 strains, mAb 53 has the potential to be applied in the development of a rapid EV71 diagnostic kit.

## Materials and Methods

### Viruses growth and purification

EV 71 strains and CA 16 strain (Accession No. U05876) are listed in Table 1. Other Enterovirus strains (B3-AF376093, C3- AY125973, C4- EU703813) were generated using the human RNA polymerase I reverse genetics system [37]. These viruses were propagated in RD cells in DMEM (Gibco, USA) supplemented with 10% fetal bovine serum (FBS). Virus activity was tested on RD cells by an end point dilution assay to determine the TCID_50_ [38] and inactivated by binary ethyleneimine (BEI) as described by Bahnemann [39], [40]. Virus was concentrated ten-fold by ultracentrifugation.

### Mice immunization, generation and screening of EV71-specific hybridoma cell line

Three SPF BALB/c mice were immunized with inactivated virus strain NUH0083-B5 in 0.1 ml of PBS, emulsified with adjuvant (Seppic, France) at a 1∶1 ratio. Mice were subjected to two boosters at 14-days intervals. Mice were euthanized three days after subjecting to a final booster intraperitoneally, and. spleen cells were harvested and fused with SP2/0 myeloma cells as described previously [41]. The hybridoma cells were cultured in DMEM with 20% FBS containing HAT or HT for 10 days. Hybridoma cells were subjected to screening by IFA, and positive clones secreting EV71-specific monoclonal antibodies were subcloned and cultured. All animal experiments were carried out in accordance with the guides for Animal Experiments of the National Institute of Infectious Disease (NIID). Experiment protocols were reviewed and approved by Institutional Animal Care and Use Committee of the Temasek Life Sciences Laboratory (Project Approval No. TLL-10-017 & TLL-11-014), National University of Singapore, Singapore.

### Antibody detection by indirect immunofluoresence assay

Vero African green monkey kidney cells were used for antibody screening. Cells were seeded overnight onto 96-well microtiter plates and infected with EV71. Upon observation of CPE after 48 h at 37°C, cells were fixed with 4% paraformaldehyde (pH 7.4) for 20 min and permeabilized with 0.1% Triton-X/PBS for 5 min. Cells were blocked with 5% FBS/PBS for 30 min, washed and incubated in hybridoma cell supernatant or primary antibody solution for 1 h followed by incubation in FITC-coupled secondary antibodies for 1 h in room temperature. Cells were washed in 0.1% Tween/PBS for thrice for 5 min each in between steps. Results were documented with an inverted microscope (Olympus) with Nikon ACT-1 software. The immunoglobulin isotype was determined using a mouse monoclonal antibody isotyping kit (Santa Cruz).

### Western blot analysis

The total proteins of EV71 strains and recombinant proteins of EV71 capsid proteins (VP1 and its fragments, VP2 and VP3) were used to analyze the immunoreactivity of the monoclonal antibodies. Proteins were resolved by SDS-PAGE and electro-blotted onto nitrocellulose membranes (Bio-Rad). Membrane were blocked in 5% blotting grade milk/PBS, and incubated in hybridoma cell supernatant or primary antibody solution for 1 h, followed with incubation in horse-radish-peroxidase coupled secondary antibody at 1∶10,000 dilution (Dako Cytomation) for another hour. The membranes were subjected to three washes for 5 min each in 0.1% Tween-20/PBS in between steps and developed with Amershem ECL Plus Western blotting detection reagents (GE Healthcare).

### Construction of GST fusion proteins incorporating VP1 fragments for epitope mapping

For obtaining the template sequence for expression of VP1 capsid protein of the B5 (NUH0083) strain, RNA was extracted directly from the supernatant of infected RD cells using RNeasy Mini Kit (QIAGEN) according to standard protocols. RT-PCR was performed to generate cDNA of the VP1 sequence using VP1-specific reverse primer (VP1-Xhol-R) and AMV reverse transcriptase (Roche applied science). Fragments of VP1 capsid proteins were expressed for characterization of monoclonal antibodies. PCR were carried out with the panel of primers in Table 2, using VP1 cDNA as the template sequence. PCR products were subsequently digested with *Xhol I* and *BamHI* restriction enzymes, and cloned into prokaryotic expression vector pGEX-4T-1 (GE Healthcare). The recombinant plasmids were subjected to sequencing and transformed into *Escherichia coli* (*E.coli*) BL21 cells for expression of gluthathione S-transferase (GST) fusion proteins. Positive bacterial clones were induced with 0.5 mM of isopropyl-b-D-thiogalactopyranoside (IPTG). Induced cells were pelleted and resuspended in PBS buffer. Total cell lysates were subjected to western blot analysis. For expression of the specific epitope of the mAbs, the DNA sequences were incorporated within the sense primer together with the reverse primer pGEX-Aat II-R, with pGEX-4-T1 as the template.

**Table 2:** Oligonucleotides primers used for construction of recombinant plasmids.

Primers	Sequence (5′→3′)
VP1-BamH-F1	5′-CCG***GGATCC***GGAGACAGGGTGGCAGATGT
VP1-Xhol-A(1–66)-R	5′-CCG***CTCGAG***TGTCTCAATCATACTCTCAT
VP1-Xhol-B(1–132)-R	5′-CCG***CTCGAG***ATCAAAGCGCATATAGGTGA
VP1-Xhol-C(1–163)-R	5′-CCG***CTCGAG***TGGTTTAGGAGCACCAGGGG
VP1-Xhol-D(1–177)-R	5′-CCG***CTCGAG***AGGGTTTGTGGCTGTCTGCC
VP1-Xhol-E(1–208)-R	5′-CCG***CTCGAG***GTACCCGTCGTAAAACCACT
VP1-Xhol-F(1–222)-R	5′-CCG***CTCGAG***ATATTCAAGATCTTTCTCCTG
VP1-Xhol-G(1–240)-R	5′-CCG***CTCGAG***AGACCCCACGGTCCGCACCG
VP1-Xhol-H(1–260)-R	5′-CCG***CTCGAG***TGCCCTGACATGCTTCATTC
VP1-BamH-f2	5′- CCG***GGATCC***CCTCTGTCCCACCGTCTACA
VP1-Xhol-a(1–210)-R	5′- CCG***CTCGAG***CGTGGGGTACCCGTCGTAAAACC
VP1-Xhol-b(1–212)-R	5′- CCG***CTCGAG***TCCAAACGTGGGGTACCCGTCG
VP1-Xhol-c(1–214)-R	5′- CCG***CTCGAG***GTGTTCTCCAAACGTGGGG
VP1-Xhol-d(1–216)-R	5′- CCG***CTCGAG***CTGTTTGTGTTCTCCAAACG
VP1-Xhol-e(1–218)-R	5′- CCG***CTCGAG***TTTCTCCTGTTTGTGTTCTCC
VP1-Xhol-e(1–220)-R	5′- CCG***CTCGAG***AAGATCTTTCTCCTGTTTGTG
VP1-BamH-aa(210–297)-F	5′- CCG***GGATCC***TTTGGAGAACACAAACAGGAG
VP1-BamH-bb(211–297)-F	5′-CCG***GGATCC***GGAGAACACAAACAGGAGAAAG
VP1-BamH-cc(212–297)-F	5′- CCG***GGATCC***GAACACAAACAGGAGAAAGATC
VP1-BamH-dd(213–297)-F	5′- CCG***GGATCC***CACAAACAGGAGAAAGATCTTG
VP1-BamH-ee(214–297)-F	5′-CCG***GGATCC***AAACAGGAGAAAGATCTTGAATATGG
VP1-BamH-ff(215–297)-F	5′- CCG***GGATCC***CAGGAGAAAGATCTTGAATATGG
VP1-BamH-gg(216–297)-F	5′- CCG***GGATCC***GAGAAAGATCTTGAATATGGAGCG
VP1-BamH-hh(217–297)-F	5′- CCG***GGATCC***AAAGATCTTGAATATGGAGCGTG
VP1-Xhol-R	5′- CCG***CTCGAG***AAGGGTAGTAATAGCGGTAC
VP1-BamH(epitope)-F	5′-CCG***GGATCC***(Epitope)TGACGATCTGCCTCGCG
pGEX-AatII-R	5′- ACT***GACGTC***TAAGAAACCATTATTATC
UTRF-5′	5′- TCCTCCGGCCCCTGAATG

Bold and italic letters indicate the sequence of restriction enzymes.

### Ascites production and IgM purification

Ascites fluid containing monoclonal antibodies was produced according to Current Protocol in Immunology from SPF BALB/c mice [42]. IgM from ascites fluid was purified using IgM purification kit (Thermo Scientific, USA) based on manufacturing protocol.

### Neutralizing antibody assay

Neutralization activity of monoclonal antibodies and ascites fluid samples were determined by in vitro microneutralization assay in RD cells. Two-fold serial monoclonal antibody dilutions (50 µl each) were mixed with equal volume of 200 TCID_50_ of virus, and incubated at 37°C for 1 h. The antibody-virus mixtures were then added to the wells of the microtiter plates containing RD cells. The highest dilution of monoclonal antibody that inhibited virus growth was considered as the neutralization antibody titer and was determined after incubation at 37°C for 96 h. Ascites was heated at 56°C for 30 min to inactivate complements before use. Each assay was performed independently for three times.

### Experimental design for passive protection

The animal experiments were conducted with two weeks old AG129 mice. These mice were obtained from B&K Universal (UK). They were housed and bred under specific pathogen-free conditions in individual ventilated cage. The institutional (NUS) guidelines for animal care and use were strictly followed (Project Approval No. IACUC-070/10(A2)11). To test the efficacy of the antibody, these mice were randomly divided into two groups of 10 mice each. Group 1 mice (prophylactic group) were injected i.p. with the purified mAb 51 antibody (0.1 ml in 50% glycerol dissolved in PBS) at a concentration of 10 µg/g of body weight one day whereas group two mice (isotype control group) was given an equal amount of purified mouse IgM as isotype control (eBioscience, San Diego, CA, USA). These two groups of mice were subjected to lethal challenge with 10^7^ plaque forming units (PFU) of EV71 strain HFM 41 (5865/SIN/00009) via the i.p. route [0.4 ml in PBS], 24 h post-injection of the immunoglobulins. Survival rates and clinical scores of the mice were monitored daily. Total limb paralysis was used as criterion for early euthanasia.

### Quantitative reverse transcription polymerase chain reaction

Spinal cord tissues from isotype control IgM-treated mice and prophylactically protected mice were homogenized for total RNA extraction by TRIzol reagent (Invitrogen). Extracted RNA (1 µg) was used for qRT-PCR using the QuantiFast SYBR Green RT-PCR kit (Qiagen, Hilden, Germany) according to the manufacturer's protocol. 5′ UTR specific primers, i.e UTRF-5′ and UTR-R-5′ (Table 2), were used for amplification. The qRT-PCR thermal cycling conditions were applied at an initial incubation at 50°C for 12 min (reverse transcription), 95°C for 6 min (initial PCR activation step), followed by 40 cycles each of 95°C for 12 s (denaturation), 60°C for 30 s (combined annealing and extension), and 77°C for 15 s. Melting curve data were collected from 50–95°C at a ramping rate of 1°C/5 s, before finally cooling at 40°C. The reaction was carried out using a Rotor-Gene Q real time PCR cycler (Qiagen). The relative expression values were normalized to the expression value of the β-tubulin housekeeping gene. Serial ten-fold dilutions of recombinant plasmid DNA containing full-length EV71 genome were included to generate a standard curve for quantitative analysis.

### Histopathologic and immunochemical analysis

Samples of spinal cords were collected, fixed in formalin, embedded in paraffin blocks cut at 5 µm thickness (Leica Autocut microtome model 2255, Leica Microsystems, Wetzler, Germany), and attached to glass slides coated with poly-L-lysine. Slides were subjected to staining with hematoxylin and eosin (H&E). In addition, immunohistochemistry (IHC) technique was used to detect the presence of viral antigen using mAb 53 and 4B12. The mAb 4B12 recognizes the 3D polymerase of EV71 virus (unpublished data). Sides were de-paraffinized with Histo-clear II (National Diagnostics, Georgia, USA), and rehydrated in sequentially graduated ethanol baths. Slides were subjected to treatment according to manufacturer's instructions (DAKO Animal research kit).

### Computational and sequence analyses

The identified epitope was used as the query sequence and subjected to BLAST analysis against all enterovirus entries in the National Center of Biotechnology Information database by the protein-protein BLAST search. Amino acid sequences were aligned by DNASTAR MegAlign using the Clustal method.
